# Assessing the Effect of Simultaneous Exposure to Noise and Cigarette Smoke on Workers’ Blood Pressure

**Published:** 2016-11

**Authors:** Farzane Rahimpour, Ehsan Rafiei Manesh, Lida Jarahi, Saba Eghbali

**Affiliations:** 1 *Department of Occupational Medicine, Mashhad University of Medical Sciences, Mashhad, Iran.*; 2 *Department of *Community Medicine*, Mashhad University of Medical Sciences, Mashhad, Iran.*

**Keywords:** Hypertension, Manpower, Noise, Smoking

## Abstract

**Introduction:**

Noise, as the most common pollutant in the industrial environment, can lead to hearing loss and negatively affect other organs such as the cardiovascular system. Cigarette smoking is a popular habit among some workers, and can also have a negative effect on the cardiovascular system. This study was aimed to investigate the effect of simultaneous exposure to noise and cigarette smoke on the blood pressure of workers at a manufacturing factory.

**Materials and Methods:**

This cross-sectional study enrolled 604 workers at a steel factory. Information relating to workers’ demography, employment, and risk factors were recorded. Based on the level of smoking per day, workers exposed to noise fell into one of the four following groups: 1) Non-smokers exposed to noise <85 DB; 2) Smokers exposed to noise <85 DB; 3) Non-smokers exposed to noise ≥85 DB; 4) Smokers exposed to noise ≥85 DB. A t-test, analysis of variance (ANOVA), and logistic regression were applied for analysis using SPSS v11.5.

**Results:**

The prevalence of hypertension, cigarette smoking, and exposure to noise ≥85 DB was 11.6%, 15.3%, and 56.4%, respectively, among the workers. The mean systolic blood pressure (SBP) and diastolic blood pressure (DBP) were 112.3 and 73.9 mmHg, respectively. A significant difference was observed between systolic and diastolic blood pressures in four groups (P=0.001). Posthoc test showed a significant difference between groups 1 and 3 (P=0.001). Regression analysis indicated no significant difference in workers who were simultaneously exposed to noise and cigarette smoke.

**Conclusion:**

This study demonstrates that noise is an important factor in terms of hypertension, with no significant differences observed in the prevalence of hypertension between workers who were simultaneously exposed to noise and cigarette smoke. It is suggested that workers’ blood pressure should be regularly monitored in noisy environments.

## Introduction

As the most common pollutant in industrial environments, noise can lead to hearing loss and many other negative effects on functions such as the cardiovascular system. Various studies have shown that environments with a noise level above 85 DB could negatively increase blood pressure among workers (1–6). It seems that higher noise levels cause greater secretion of adrenaline, leading to vasoconstriction, stress, and raised blood pressure. 

On the other hand, noise changes the heart beat, decreases heart output, and increases respiratory rate (7–9). 

Because the presence of high blood pressure in a noisy environment could in the long term cause chronic hypertension, investigating the relationship between high blood pressure and exposure to noise is of considerable importance (10).

Up to 2009, the prevalence of tobacco smoking was approximately 26% in Western countries (11). There are currently 1.3 billion smokers, 84% of whom live in developing countries. It is predicted that the death rate because of smoking will increase to 8 million people by 2030, especially in countries whose economic growth is relatively low (12). Nicotine, as a stimulant of the sympathetic nervous system, acutely raises heartbeat, blood pressure, and myocardial contractility (13), while its chronic effects such as arterial stiffness persist for a decade after giving up smoking (14). High blood pressure could be considered as one of the most common causes of death associated with cardiovascular diseases and smoking (15).

The association between high blood pressure and smoking is still under investigation. Some studies have demonstrated that blood pressure increases in smokers (16,17), especially those who smoke more than 15 cigarettes per day (15, 18) and who are older (17). However, other studies have shown a lower blood pressure among smokers in comparison with non-smokers (17,19).

 Cotinine, the main metabolite of nicotine, is a vasodilator and could, therefore, decrease blood pressure (20). For these reasons and because of the sparsity of previous studies examining the effect of simultaneous exposure to noise and cigarette smoke, the present study intends to examine the effect of this combination on the blood pressure of workers in manufacturing companies.

## Materials and Methods

This study was a cross-sectional survey carried out in a steel factory in Khorassan Province over a period from 2014 to 2015. All 604 participants were male and had more than 1 year of work experience, and all knowingly contributed to the research. Exclusion criteria in this study were a diagnosis of hypertension, diabetes, chronic cardiovascular, or kidney diseases, and use of drugs such as corticosteroids which affect blood pressure before beginning employment at the factory. After exclusion of 17 workers diagnosed with diabetes, three with chronic kidney disease, one taking corticosteroids, and three presenting with high blood pressure, 580 workers were included in the final analysis. This study was approved by the Ethics Committee of Mashhad University of Medical Sciences (code: 930365).

Required demographic, medical, and employment data were assembled through interviews. Demographic data consisted of age, gender, body mass index (BMI), marital status, educational degree, medical history (previous diagnosis of hypertension, diabetes, heart disease, high blood pressure in first-degree relatives and use of specific drugs), and lifestyle (high and low salt consumption). As the workers included made no regular use of hearing protection devices, this factor was not assessed. In this study, the term exercise refers to regular 30-minute sessions of exercise, 3 days per week (21); smokers are defined as those with a history of smoking more than 100 cigarettes (five packets)up to now; otherwise they are considered non-smokers (2). Variables relating to employment (work station, work experience in the current station, work shift, level of exposure to noise in the current station) were recorded by the Health and Safety team of the factory. The noise level was measured by this team at the different work stations. Sound pressure was determined using a dosimeter device (CEL-440), with a range of 20–140 DB. In this study, noises ≥85 DB were prohibited.

The heights and weights of the workers were measured, and their BMI was calculated by dividing their weight by the square of their height. Blood pressure was measured using 10–1240cm cuffs from ALPK2. SBP and DBP were measured twice, at least 30 minutes after eating, exercising, or smoking (22) twice a day (5 minutes after sitting down) from the workers’ right hand, and the average values were recorded. Hypertension was defined as an average SBP ≥140 mmHg at rest or an average DBP ≥90 mmHg, or was recorded in workers being treated for high blood pressure after being employed (22).

Finally, participants were classified into four groups on the basis of data collected with respect to their level of exposure to noise and cigarette smoke, as follows: 1) Non-smokers exposed to noise <85 DB; 2) Smokers exposed to noise <85 DB; 3) Non-smokers exposed to noise ≥85 DB; 4) Smokers exposed to noise ≥85 DB.

Mean values, standard deviation (SD), and range are presented for quantitative variables. A t-test and analysis of variance (ANOVA) were used for comparison of the variables in each group. A Chi-square test was utilized for comparing qualitative variables. Logistic regression analysis was used to eliminate confounding variables and assess the relationship between exposure to noise and smoke and hypertension. All tests showing a confidence level equal to 95% and a significance level less than 0.05 were considered significant. All statistical calculations were conducted using SPSS version 16.

## Results

The research sample in this study consisted of 580 workers at a steel factory. The average age was 39.1 (21–64) years, the average BMI was 24.9 (16.4–36.6) kg/m^2^, the average work experience was 12.4 (2–24) years, and the average SBP and DBP were 112.3 (70–180) and 73.9 (40–105) mmHg, respectively. Table 1 shows the prevalence of the main variables in the studied population. In total, 228 workers (39.3%) were non-smokers exposed to noise <85 DB (Group 1), 25 (4.3%) were smokers exposed to noise <85 DB (Group 2), 263 (45.3%) were non-smokers exposed to a prohibited noise ≥85 DB (Group 3), and 64 (11%) were smokers exposed to a prohibited noise ≥85 DB (Group 4).

Table 2 shows a comparison of demographic features and risk factors for hypertension using an ANOVA test for the three variables of age, work experience, and BMI. A Chi-square test was utilized to compare the workers in the four groups who consumed large amounts of salt and regularly exercised. There was no significant difference between average work experience, BMI, and the consumption of a salty diet among the four groups (P≥0.05), but there was a significant difference in terms of the average age (P=0.036) and participation in regular exercise (P=0.001) among the four groups.

An ANOVA test was applied to examine the average SBP and DBPs (Table.3). A significant difference was observed in the average SBP (P=0.001) and DBP (P=0.001). A post hoc test showed a significant difference between Groups 1 and 3 in terms of SBP and DBP. The mean SBP and DBP in smokers who were exposed to prohibited noise levels (Group 4) was approximately 2.6 mmHg and 3.7 mmHg greater than that of non-smokers who were exposed to noise <85 DB (Group 1).

A chi-square test was utilized to assess the prevalence of systolic and diastolic hypertension in the four groups (Table 3). Although the prevalence of hypertension in Groups 3 and 4 was considerably larger than that in Groups 1 and 2, this difference was not statistically significant (P=0.184). The highest prevalence of high blood pressure and systolic and diastolic hypertension was observed among non-smokers who were exposed to the prohibited level of noise (Group 3). Logistic regression analysis was used to verify the assessment of relationships between noise exposure, smoke exposure, and hypertension and also to control for confounding variables. Blood pressure was regarded as a dependent variable, and the effects of confounding independent variables such as age and regular exercise were compared in order to find any association with cigarette smoke and noise (Tables 4 and 5). This analysis did not reveal any significant differences between the frequency of systolic and diastolic hypertension, while a significant difference was seen between age and hypertension.

**Table 1:** Total prevalence of exposure to noise ≥85DB, cigarette smoking, and hypertension in participants

	**Exposure to noise ≥85DB**	**Cigarette smoking**	**Diagnosed with hypertension** *
Positive Number (percentage)	327 (56.4%)	89 (15.3%)	67 (11.6%)
Negative Number (percentage)	253 (43.6%)	491 (84.7%)	513 (88.4%)

* 34 workers were diagnosed with systolic hypertension (5.9%) and 55 workers with diastolic hypertension (9.5%).

**Table 2:** Comparison of demographic variable in study groups

	**Group 1**	**Group 2**	**Group 3**	**Group 4**	**P-Value**
Age (year); mean (SD)	38.2 (7.5)	44 (8.5)	38.1 (6.7)	44.2 (6.4)	0.036*
Work experience (year); mean (SD)	12.8 (5.5)	13.0 (6.4)	11.6 (5.5)	14.1 (5.4)	0.182*
BMI (kg/m^2^); mean (SD)	25.2 (3.8)	23.4 (3.2)	25.0 (3.7)	24.6 (4.1)	0.468*
Salty diet; % (n)	44% (102)	44% (11)	48% (127)	56% (46)	0.415**
Regular exercise; % (n)	36.8% (84)	40% (10)	5.7% (15)	3.1% (2)	0.001**

BMI: body mass index; SD: standard deviation

* ANOVA

** Chi-square

*** Non-smokers exposed to noise <85 DB (Group 1); Smokers exposed to noise <85 DB (Group 2); non-smokers exposed to noise ≥85 DB (Group 3); smokers exposed to noise ≥85 DB (Group 4)

**Table 3:** Comparing the prevalence of hypertension, systolic and diastolic hypertension, and mean SBP and DBP among study groups

	**Group 1** ** (227)**	**Group 2 ** **(25)**	**Group 3 ** **(263)**	**Group 4 ** **(64)**	**P-Value** *
Hypertension; n (%)	19 (8.3%)	2(8%)	38 (14.4%)	8 (12.5%)	0.184*
Systolic hypertension; n (%)	10 (4.4%)	1(4%)	21 (8%)	2 (3.1%)	0.254*
Diastolic hypertension; n (%)	14 (6.1%)	1(4%)	34 (12.9%)	6 (9.4%)	0.058*
Mean (SD) SBP	109.3 (0.93)	111/2(2/9)	113/8 (0/8)	111/9 (1/6)	0.001**
Mean (SD) DBP	70.5 (0.6)	71/8(2/0)	76/0 (0/5)	74/2 (1/2)	0.001

DBP: Diastolic blood pressure; SBP: systolic blood pressure; SD: standard deviation

* Chi-square test

** ANOVA test

*** Non-smokers exposed to noise <85 DB (Group 1); Smokers exposed to noise <85 DB (Group 2); non-smokers exposed to noise ≥85 DB (Group 3); smokers exposed to noise ≥85 DB (Group 4)

**Table 4:** Regression results and OR for prediction systolic hypertension in study groups in the presence and absence of the meaningful confounding variables (age and regular exercise of participants)*

	**Variable**	**B**	**SE**	**p-value**	**OR**	**95% CI for OR**	
						**Lower**	**Upper**
**Systolic hypertension**	Group 1			0.271			
	Group 2	0.096	1.071	0.928	1.101	0.135	8.976
	Group 3	0.256	1.248	0.828	0.774	0.067	8.938
	Group 4	0.734	1.046	0.483	2.083	0.268	16.169
**Systolic **	Group 1			0.037			
**hypertension**	Group 2	0.782	1.108	0.480	2.187	0.249	19.180
	Group 3	0.149	1.290	0.908	1.160	0.093	14.543
	Group 4	1.710	1.104	0.121	5.528	0.635	48.127
	Age	0.100	0.026	0.001	1.106	1.050	1.164
	Regular exercise	0.447	0.526	0.395	0.639	0.228	1.791

CI: confidence interval; OR: Odds ratio; SE: standard error

* Non-smokers exposed to noise <85 DB (Group 1); Smokers exposed to noise <85 DB (Group 2); non-smokers exposed to noise ≥85 DB (Group 3); smokers exposed to noise ≥85 DB (Group 4)

**Table 5:** Regression results and OR for prediction diastolic hypertension in study groups in the presence and absence of the meaningful confounding variables (age and regular exercise of participants)*

	**Variable**	**B**	**SE**	**p-value**	**OR**	**95% CI for OR**	
						**Lower **	**Upper **
**Diastolic hypertension**	Group 1			0.068			
	Group 2	0.451	1.057	0.670	1.570	0.198	12.470
	Group 3	0.909	1.107	0.411	2.483	0.284	21.740
	Group 4	1.271	1.037	0.220	3.563	0.467	27.201
**Diastolic hypertension**	Group 1			0.038			
	Group 2	0.737	1.074	0.493	2.089	0.255	17.132
	Group 3	1.032	1.128	0.360	2.807	0.308	25.590
	Group 4	1.648	1.065	0.122	5.196	0.644	41.930
	Age	0.048	0.021	0.021	1.049	1.007	1.093
	Regular exercise	0.344	0.462	0.457	1.410	0.570	3.491

CI: confidence interval; OR: Odds ratio; SE: standard error

* Non-smokers exposed to noise <85 DB (Group 1); Smokers exposed to noise <85 DB (Group 2); non-smokers exposed to noise ≥85 DB (Group 3); smokers exposed to noise ≥85 DB (Group 4)

## Discussion

This study examined the effect of simultaneous exposure to noise and cigarette smoke on workers’ blood pressure. The observed difference in systolic and diastolic hypertension was statistically significant, but not clinically significant. This observation is consistent with the findings of Chang et al. who conducted a prospective cohort study in Taiwan over a 10-year period (23).

In the current study, a significant difference was observed between mean systolic and diastolic hypertension in Groups 1 and 3 comparing those exposed and not exposed to noise. This shows that prohibited noise levels could negatively increase the prevalence of systolic and diastolic hypertension, although no synergic effect was observed in the concurrency of noise and cigarette smoke. In another study reported by Sancini et al., a significant increase was seen in average SBP and DPB in the group exposed to noise (2); consistent with the findings of the present study. Another study by Lee et al. showed a relationship between continuous exposure to noise and systolic hypertension (24). Rizi et al. (2013) found a significant difference in systolic hypertension in 80 workers in industrial environments in Isfahan. However, this significant difference was not observed in terms of diastolic hypertension (1). The significant difference in the study by Rizi may be due to the younger age of the studied population (age average, 26.6 years) in comparison with the present study. Talbott et al. conducted a study in which there was a strong relationship between hearing loss and diastolic hypertension, while no significant relationship was observed between blood pressure and exposure to noise (4). This heterogeneity could be due to various methods of measuring blood pressure being used, as well as restrictions on characteristics and confounding variables.

The greatest prevalence of high blood pressure and systolic and diastolic hypertension was observed among non-smokers who were exposed to the prohibited level of noise (Group 3). Logistic regression analysis was applied to assess the relationship between prevalence of hypertension in the presence of the confounding variables of age and regular exercise in the four groups. This analysis did not indicate any significant differences between systolic and diastolic hypertension among the groups. Previous research in 1,205 workers in the textile industry revealed a prevalence of hypertension that was 1.34 times greater than that in the control group (25). This difference may be due to higher noise levels. Gan et al. reported that people exposed to continuous noise at a prohibited level may be diagnosed with diastolic hypertension two or three times more frequently than controls. In comparison with those who have never been exposed to continuous prohibited noise, this relationship is mostly observed in smokers (5). Other reasons for the differences in the studies of Panch et al. and Gan et al. may include their larger target populations (5,25). Logistic regression analysis in the current study demonstrated no significant difference between the groups in terms of diagnosis of systolic or diastolic hypertension in the presence of the confounding variables of age and regular exercise. However, older workers may face a 1.1× greater risk of systolic hypertension and 1.04× greater risk of diastolic hypertension compared with younger workers. This observation is consistent with the findings of Boudin et al. (2009) who concluded that the effect of noise on blood pressure based on various age groups was significant (26). Due to the existence of few smokers in this study, the authors could not classify the research sample into various age groups. Zhang et al. assessed the same relationship between diastolic hypertension and age (27).

Aziz et al. examined the relationship between smoking and high blood pressure in 712 males and females and found no significant relationship between the prevalence of hypertension and smoking, which was consistent with the results of the present study (28), Although it has not been clearly proven yet, cotinine, as the vasodilator and the main metabolite of nicotine, could decrease blood pressure (21). Lee et al. investigated the effect of giving smoking up on hypertension and found that those who gave it up for more than 1 year were diagnosed with higher systolic and diastolic hypertension in proportion to the people who were still smoking; however its precise mechanism is not clear (29). Leone et al. showed a temporary relationship between smoking and high blood pressure which may become chronic due to the atherogenesis effect of smoking (30). These findings were inconsistent with the findings of the present study, although this difference may be due to the different characteristics of the target population.

In the current study, no significant relationship was observed between simultaneous exposure to noise and high blood pressure, and no significant association was seen between being exposed to the prohibited level of noise and smoking and high blood pressure after controlling for confounding variables. This may be due to the insufficient number of smokers in the population in this study. One of the limitations of this study is its subjective nature, since workers may not be totally honest due to the culture dominant in the society which discourages smoking, alcohol intake, and using other forms of smoking such as opium. Another limitation was the cross-sectional design, which did not allow investigation of the association between smoking and being exposed to noise. Innovation could be considered one of the strengths of this study, since the effect of simultaneous exposure to noise and cigarette smoke on blood pressure has not been examined so far. It is suggested that a longitudinal study is carried out on larger sample sizes.

## Conclusion

Although no association was found between smoking and being exposed to noise, this may be due to the insufficient number of smokers in the study population. However, a relationship between noise and hypertension was observed, and it is therefore suggested that a longitudinal study is carried out on larger sample sizes.
